# Cadmium body burden and health effects after restoration of cadmium-polluted soils in cadmium-polluted areas in the Jinzu River basin

**DOI:** 10.1265/ehpm.23-00132

**Published:** 2023-09-09

**Authors:** Masaru Sakurai, Yasushi Suwazono, Kazuhiro Nogawa, Yuuka Watanabe, Miyuki Takami, Yasumitsu Ogra, Yu-Ki Tanaka, Hirotaro Iwase, Kayo Tanaka, Masao Ishizaki, Teruhiko Kido, Hideaki Nakagawa

**Affiliations:** 1 Department of Social and Environmental Medicine, Kanazawa Medical University, Uchinada 920-0293, Japan; 2 Department of Occupational and Environmental Medicine, Graduate School of Medicine, Chiba University, Chiba 260-8670, Japan; 3 Graduate School of Pharmaceutical Sciences, Chiba University, Chiba 260-8675, Japan; 4 Department of Legal Medicine, Graduate School of Medicine, Chiba University, Chiba 260-8670, Japan; 5 School of Health Sciences, Kanazawa University, Kanazawa 920-0942, Japan

**Keywords:** Urinary cadmium, β2-microglobulin, NAG, Restoration of cadmium-polluted soils, The Jinzu River basin

## Abstract

**Background:**

*Itai-itai* disease is caused by environmental cadmium (Cd) pollution in the Jinzu River basin in Japan. To reduce the Cd contamination of rice, soil restoration of paddy fields was carried out. We evaluated the effect of soil restoration on the health status of residents of the former Cd-polluted area.

**Methods:**

Participants were 1,030 men and 944 women who lived in the area of restoration of Cd-polluted rice paddies. First morning urine was collected and urinary Cd, β2-microglobulin (β2MG), and N-acetyl-β-D-glucosaminidase (NAG) levels were measured. Associations among age, years of residence before and after soil restoration, and urinary Cd, β2MG, and NAG levels were evaluated by multiple regression analysis.

**Results:**

The geometric mean (interquartile range) of urinary Cd (µg/g Cr) was 1.00 (0.58–1.68) in men and 1.67 (1.02–2.91) in women. The geometric means of urinary β2MG (µg/g Cr) and NAG (U/g Cr) were 174.6 (92.6–234.2) and 1.47 (0.72–3.14) in men, and 217.6 (115.3–28.7) and 1.48 (0.73–2.96) in women, respectively. Urinary Cd, β2MG, and NAG were significantly positively correlated (p < 0.01 all). Age and duration of residence in the Cd-polluted area before soil restoration were independently associated with urinary Cd, β2MG, and NAG. Among the 916 participants who had resided in the area before the soil restoration, urinary Cd concentrations were significantly higher, thus by 1.03-fold (95% CI, 1.01–1.04) in men and 1.03-fold (95% CI, 1.01–1.05) in women, when the years of residence before soil restoration by each 5-years increment. By contrast, urinary Cd concentrations were significantly lower, thus 0.97-fold (95% CI, 0.96–0.99) lower in men and 0.97-fold (95% CI, 0.95–0.99) lower in women, by each 5-year increment of residence after soil restoration. A similar association was observed for urinary β2MG concentration, and no significant association was observed for urinary NAG levels in men or women.

**Conclusions:**

Cd exposure and associated renal tubular dysfunction in residents of a former Cd-polluted area were influenced by Cd exposure from the environment prior to soil restoration. Soil restoration in Cd-polluted areas reduced the Cd exposure of local residents.

## Introduction

*Itai-itai* disease has occurred in the Jinzu River basin since the Taisho Era, causing osteomalaecia with severe bone pain [1, 2]. Cadmium (Cd) released from a mine upstream polluted rice-field soils. More than 10,000 people live in this Cd-polluted area and many of them have kidney damage and proximal renal tubular dysfunction. A research group of the Ministry of the Environment conducted a health survey between 1976 and 1984 on 12,559 residents of Cd-polluted areas in eight prefectures in Japan; 333 residents were suspected to have proximal tubular dysfunction and 202 were reported to have proximal tubular dysfunction [3]. Almost all of these 202 persons were residents of the Jinzu River basin, which is the largest Cd-polluted area in Japan.

Negative effects on health associated with environmental and occupational exposure to heavy metals such as Cd have been widely reported. Excessive exposure to environmental Cd triggers renal tubular dysfunction and abnormal bone metabolism, and increases the risk for all-cause mortality [4, 5] and cause-specific mortalities from cancer [6, 7] and renal [4, 5, 8, 9], cardiovascular [10–13], respiratory [13, 14], and digestive [13, 15] diseases. Cd exposure is associated with lifestyle-related diseases including hypertension [16, 17] and diabetes [18]. In recent years, Cd-polluted areas have become widespread in countries such as China and Thailand, associated with negative effects on human health [19–22]. Heavy metal exposure affects the health status of the next generation; children exhibit increased morbidity [23, 24], more congenital defects [23–25], and more neurobehavioral and cognitive dysfunctions [24].

To reduce the Cd concentration in rice cultivated in the Jinzu River basin, soil restoration was carried out in Toyama Prefecture. In 1963, a water supply from uncontaminated water sources was installed in some areas of the Jinzu River basin. In addition, based on a 1970 law, the Cd concentration in rice paddy soil and brown rice was evaluated from 1971 to 1976, and about 1,500 ha of paddy fields were designated as areas requiring soil contamination improvement. Construction work was carried out in these areas from 1979 to 2012, restoring 863 ha of Cd-polluted paddy fields and converting the rest of the land into commercial and residential areas. As a result of the soil restoration, the Cd concentration in rice decreased to the same level as in Cd-non-polluted areas [26]. However, the health status of the residents of the Cd-polluted area have not been investigated.

It is important to evaluate the effect of soil restoration on the health status of local residents in response to large-scale Cd contamination in Japan to resolve the problem of environmental Cd pollution. Therefore, we evaluated the health status of residents of the former Cd-polluted area and the effectiveness of soil restoration in the Jinzu River basin.

## Methods

### 1. Participants

The participants were residents of a former Cd-polluted area that underwent soil restoration in the Jinzu River basin in Toyama Prefecture, Japan. We held an explanatory meeting about the survey for the general residents of six districts, including areas where soil restoration was carried out. After the meeting, community representatives sent the research plan to all households, and approximately 20% of such households indicated that they would participate. A total of 2,118 participants (1,105 men and 1,013 women) aged ≥20 years offered to participate in the survey, 1,974 (93.2%) of whom participated in health surveys conducted in 2020 and 2021. In the analysis of the relationships between years of residence and urinary variables, 44 participants who had incomplete questionnaire data regarding their residence history were excluded. Thus, 1,930 participants were included in the analysis, 916 (519 men and 397 women) of whom were living at their current address before the soil restoration.

### 2. Data collection

A urine collection container and questionnaire were mailed to the participants. First morning urine was collected from the participants. Participants submitted their specimens to the community center, and they were collected by researchers during the morning of the same day. Urine specimens were pH-adjusted at the laboratory and stored frozen. Samples were used to measure urinary Cd, β2-microglobulin (β2MG), N-acetyl-β-D-glucosaminidase (NAG) and creatinine (Cr). Measurement of Cd was performed by inductively coupled plasma mass spectrometry (ICP-MS). The urinary β2MG concentration was determined by a latex agglutination method, and the urinary NAG concentration was determined by colorimetric assay (BML Inc, Tokyo, Japan).

In the questionnaire survey, sex, age, history of residence, history of farming, history of eating home-grown rice and vegetables, history of drinking river water, and smoking status were investigated. The cumulative years of residence before and after soil restoration were calculated from the history of each residence and the year of soil restoration completion. For participants who lived outside the target area for soil restoration, the years of residence before soil restoration was defined as 0 years.

### 3. Statistical analysis

For urinary Cd, β2MG and NAG, Cr-corrected values were used for the analysis [27]. We evaluated the distribution of these urinary markers by age and years of residence in the Cd-polluted area before soil restoration. The variables were log-transformed for the analysis, and the geometric mean and interquartile range were calculated. The associations among age, duration of residence before soil restoration, and urinary indices of Cd exposure were determined by multiple regression analysis. Based on the β coefficient and standard error (SE), we calculated the increase in log-transformed urinary indices (95% confidence interval [CI]) per 5-year increase in age and residency. Similarly, the relationships between years of residence before and after soil restoration and each urinary index were evaluated by multiple regression analysis for 916 participants who were living at their current address before the soil restoration. Multivariate adjustments were made for age, years of residence, and smoking status (never, ex-smoker, or current smoker).

### 4. Ethical considerations

Written informed consent was obtained from the participants. This study was approved by the Ethics Committee of Kanazawa University (No. 968-I) and the Ethics Committee of Kanazawa Medical University (Nos. I 484 and I 750).

## Results

The distribution of the urinary markers according to Cd exposure is shown in Table 1. The geometric mean (interquartile range) of urinary Cd (µg/g Cr) was 1.00 (0.58–1.68) in men and 1.67 (1.02–2.91) in women. The geometric means of urinary β2MG (µg/g Cr) and NAG (U/g Cr) were 174.6 (91.6–234.2), 1.47 (0.72–3.14) in men, and 217.6 (115.3–278.7), 1.48 (0.73–2.96) in women, respectively. Urinary Cd, β2MG, and NAG were significantly positively correlated with each other (Table 2). The geometric mean for each marker was higher for older men and women (p < 0.001) (Table 1).

**Table 1:** Urinary markers of residents of the Jinzu River basin after Cd-contaminated soil restoration.

			**Men**				**Women**			
	
			**N**	**Geometric** **mean**	**Interquartile** **range**	**P for** **trend**	**N**	**Geometric** **mean**	**Interquartile** **range**	**P for** **trend**
Urinary Cd (µg/g Cr)	Total		1030	1.00	(0.58–1.68)		944	1.67	(1.02–2.91)	

	Age (y)	≤29	34	0.33	(0.24–0.42)	<0.001	51	0.42	(0.28–0.60)	<0.001
		30–39	81	0.41	(0.29–0.57)		73	0.71	(0.45–1.07)	
		40–49	147	0.55	(0.37–0.77)		117	0.91	(0.61–1.38)	
		50–59	154	0.84	(0.55–1.19)		144	1.53	(1.05–2.22)	
		60–69	268	1.22	(0.90–1.77)		251	2.25	(1.66–3.19)	
		70–79	269	1.55	(1.17–2.18)		209	2.49	(1.86–3.55)	
		≥80	77	1.90	(1.41–2.53)		99	2.99	(2.16–4.22)	

Urinary β2MG (µg/g Cr)	Total		1030	174.6	(91.6–234.2)		944	217.6	(115.3–278.7)	

	Age (y)	≤29	34	95.7	(65.6–118.8)	<0.001	51	89.5	(73.3–119.5)	<0.001
		30–39	81	93.9	(69.9–120.8)		73	107.9	(80.2–152.7)	
		40–49	147	105.0	(77.7–130.9)		117	110.3	(83.5–144.2)	
		50–59	154	135.1	(85.0–165.9)		144	160.9	(110.1–207.9)	
		60–69	268	164.5	(94.7–257.9)		251	198.5	(133.3–268.8)	
		70–79	269	233.1	(122.4–369.7)		209	294.1	(167.3–400.0)	
		≥80	77	865.2	(184.7–2449.5)		99	1337.2	(334.4–5173.4)	

Urinary NAG (U/g Cr)	Total		1003	1.47	(0.72–3.14)		918	1.48	(0.73–2.96)	

	Age (y)	≤29	33	0.76	(0.29–1.41)	<0.001	51	0.75	(0.39–1.06)	<0.001
		30–39	80	0.79	(0.52–1.26)		71	0.95	(0.52–1.83)	
		40–49	144	0.98	(0.49–1.86)		115	1.01	(0.66–1.58)	
		50–59	150	1.14	(0.60–2.23)		138	1.22	(0.61–2.39)	
		60–69	260	1.65	(0.94–3.44)		247	1.53	(0.79–3.05)	
		70–79	263	2.13	(1.06–4.69)		203	1.96	(0.96–3.85)	
		≥80	73	2.56	(1.20–6.05)		93	3.22	(1.61–7.48)	

Cd, cadmium; β2MG, β2-microgloblin; NAG, N-acetylglucosaminidase; Cr, creatinine.

**Table 2:** Correlation coefficients among urinary markers for residents after soil restoration.

		**log (urinary Cd)**		**log (urinary β2MG)**		**log (urinary NAG)**	
		**r**	**p**	**r**	**p**	**r**	**p**
log (urinary Cd)	Men	-		0.369	<0.001	0.368	<0.001
	Women	-		0.441	<0.001	0.349	<0.001
log (urinary β2MG)	Men			-		0.410	<0.001
	Women			-		0.492	<0.001
log (urinary NAG)	Men					-	
	Women					-	

Cd, cadmium; β2MG, β2-microgloblin; NAG, N-acetylglucosaminidase.

Table 3 shows the distribution of urinary markers by years of residence in the Cd-polluted area before soil restoration. In both men and women, the longer the period of residence before soil restoration, the higher the geometric mean of all urinary markers of Cd exposure. Multiple regression analysis was used to evaluate the relationship between the duration of residence in the Cd-polluted area before soil restoration and urinary markers (Table 4). The years of residence in Cd-polluted areas was significantly associated with urinary Cd, β2MG, and NAG, independent of age, in both men and women. When the years of residence in the Cd-polluted area longer by each-5 years, the log-transformed urinary Cd (SE) levels were higher by 0.010 (0.002) for men and 0.006 (0.003) for women; thus, urinary Cd level were higher by 1.02-fold (95% CI, 1.01–1.03) in men and 1.01-fold (95% CI, 1.00–1.03) in women by each 5-year longer in Cd-polluted area residence before soil restoration. Similarly, markers of urinary renal tubular dysfunction, except for NAG in women, were significantly and positively associated with the duration of residence in the Cd-polluted area.

**Table 3:** Urinary markers according to years of residence in the Cd-polluted area before soil restoration.

	**Years of residence in** **the polluted area**	**Men**				**Women**			
	
		**N**	**Geometric** **mean**	**Interquartile** **range**	**p for** **trend**	**N**	**Geometric** **mean**	**Interquartile** **range**	**p for** **trend**
Urinary Cd (µg/g Cr)	None ^a^	451	0.77	(0.42–1.35)	<0.001	482	1.39	(0.82–2.42)	<0.001
	≤10	69	0.73	(0.43–1.21)		89	1.26	(0.79–2.13)	
	10–19	114	1.00	(0.58–1.60)		100	1.74	(1.17–2.81)	
	20–29	103	1.17	(0.84–1.59)		106	2.04	(1.51–3.18)	
	30–39	98	1.28	(0.90–1.85)		69	2.48	(1.67–3.30)	
	40–49	88	1.65	(1.26–2.17)		52	2.72	(2.08–3.99)	
	≥50	92	1.76	(1.31–2.57)		34	3.93	(3.12–4.92)	

Urinary β2MG (µg/g Cr)	None ^a^	451	140.3	(82.9–170.9)	<0.001	482	178.8	(104.6–242.6)	<0.001
	≤10	69	144.2	(84.6–178.7)		89	152.1	(97.0–220.7)	
	10–19	114	143.1	(88.3–201.8)		100	189.5	(126.0–240.9)	
	20–29	103	169.8	(100.0–243.2)		106	207.3	(131.0–277.6)	
	30–39	98	169.5	(98.0–288.7)		69	292.0	(139.0–344.6)	
	40–49	88	300.7	(129.1–519.3)		52	587.8	(184.2–1254.0)	
	≥50	92	496.0	(146.4–1048.7)		34	1506.3	(239.6–5270.6)	

Urinary NAG (U/g Cr)	None ^a^	440	1.15	(0.55–2.23)	<0.001	470	1.38	(0.71–2.70)	<0.001
	≤10	67	1.33	(0.74–2.37)		88	1.23	(0.60–2.33)	
	10–19	112	1.27	(0.61–2.65)		98	1.32	(0.64–2.56)	
	20–29	101	1.74	(0.93–3.59)		102	1.43	(0.73–3.22)	
	30–39	95	1.88	(1.04–3.84)		68	1.89	(0.90–3.53)	
	40–49	87	2.05	(1.15–4.59)		49	2.06	(0.97–3.96)	
	≥50	88	2.73	(1.49–5.21)		33	2.83	(1.23–6.23)	

^a^ None; no history of residence in the target area for restoration of soil Cd contamination.

Cd, cadmium; β2MG, β2-microgloblin; NAG, N-acetylglucosaminidase; Cr, creatinine.

**Table 4:** Relationships between years of residence before soil restoration and variables related to Cd exposure.

		**Association with log-transformed variables ^a^**		**Increase rate of the ** **variables (95%CI)**		**P**

		**β coefficient**	**SE**			
Urinary Cd (µg/g Cr)						
Men	Age (+5 y)	0.064	0.003	1.16	(1.14–1.17)	<0.001
	Years of residence before soil restoration (+5 y)	0.010	0.002	1.02	(1.01–1.03)	<0.001
Women	Age (+5 yr)	0.070	0.003	1.17	(1.16–1.19)	<0.001
	Years of residence before soil restoration (+5 y)	0.006	0.003	1.01	(1.00–1.03)	0.015

Urinary β2MG (µg/g Cr)						
Men	Age (+5 y)	0.064	0.005	1.16	(1.13–1.19)	<0.001
	Years of residence before soil restoration (+5 y)	0.017	0.004	1.04	(1.02–1.06)	<0.001
Women	Age (+5 yr)	0.078	0.005	1.20	(1.17–1.22)	<0.001
	Years of residence before soil restoration (+5 y)	0.020	0.005	1.05	(1.03–1.07)	<0.001

Urinary NAG (U/g Cr)						
Men	Age (+5 y)	0.046	0.005	1.11	(1.09–1.14)	<0.001
	Years of residence before soil restoration (+5 y)	0.015	0.004	1.04	(1.02–1.05)	<0.001
Women	Age (+5 yr)	0.051	0.004	1.12	(1.10–1.15)	<0.001
	Years of residence before soil restoration (+5 y)	−0.002	0.004	1.00	(0.98–1.02)	0.74

^a^ Adjusted for smoking status.

Years of residence of participants living outside the target area for soil remediation work was calculated as 0 years.

Cd, cadmium; β2MG, β2-microgloblin; NAG, N-acetylglucosaminidase; Cr, creatinine; SE, standard error of the coefficient.

Among the participants who lived in the Cd-polluted area before soil restoration, the median (interquartile range) years of residence before soil restoration was 30.8 (17.2–46.2) in men and 24.5 (12.8–37.7) in women; after soil restoration, the respective values were 31.5 (22.7–42.7) in men and 40.7 (30.1–52.5) in women. Table 5 shows the relationships between years of residence before and after soil restoration and urinary markers. The urinary Cd level was significantly higher, by 1.03-fold (95% CI, 1.01–1.04) in men and 1.03-fold (95% CI, 1.01–1.05) in women, when years of residence before soil restoration longer by 5 years. In contrast, the urinary Cd level was significantly lower, by 0.97-fold (95% CI, 0.96–0.99) in men and 0.97-fold (95% CI, 0.95–0.99) in women, when years of residence after soil restoration longer by 5 years. Similar associations were observed between years of residence and urinary β2MG. Urinary NAG showed a similar tendency; however, no significant association was observed in men or women.

**Table 5:** Relationships between urinary markers and years of residence for participants living before soil restoration (n = 916).

		**Association with log-transformed variable**		**Increase rate of the variables (95%CI)**		**P**
		**β coefficient**	**SE**			
Urinary Cd (µg/g Cr)						
Men (n = 519)	Years of residence before soil restoration (+5 y)	0.011	0.003	1.03	(1.01–1.04)	0.001
	Years of residence after soil restoration (+5 y)	−0.011	0.003	0.97	(0.96–0.99)	0.001
Women (n = 397)	Years of residence before soil restoration (+5 y)	0.011	0.004	1.03	(1.01–1.05)	0.007
	Years of residence after soil restoration (+5 y)	−0.011	0.004	0.97	(0.95–0.99)	0.007
Urinary β2MG (µg/g Cr)						
Men (n = 519)	Years of residence before soil restoration (+5 y)	0.028	0.007	1.07	(1.03–1.10)	<0.001
	Years of residence after soil restoration (+5 y)	−0.028	0.007	0.94	(0.91–0.97)	<0.001
Women (n = 397)	Years of residence before soil restoration (+5 y)	0.040	0.009	1.10	(1.05–1.14)	<0.001
	Years of residence after soil restoration (+5 y)	−0.041	0.009	0.91	(0.87–0.95)	<0.001
Urinary NAG (U/g Cr)						
Men (n = 507)	Years of residence before soil restoration (+5 y)	0.007	0.007	1.02	(0.99–1.05)	0.269
	Years of residence after soil restoration (+5 y)	−0.008	0.007	0.98	(0.95–1.01)	0.254
Women (n = 385)	Years of residence before soil restoration (+5 y)	0.005	0.008	1.01	(0.98–1.05)	0.486
	Years of residence after soil restoration (+5 y)	−0.006	0.008	0.99	(0.95–1.02)	0.469

Adjusted for age and smoking status.

Cd, cadmium; β2MG, β2-microgloblin; NAG, N-acetylglucosaminidase; Cr, creatinine; SE, standard error of the coefficient.

## Discussion

We evaluated the associations among age, years of residence, and urinary Cd levels in residents of a Cd-polluted area after soil restoration. The geometric mean urinary Cd concentration was 1.00 µg/g Cr for men and 1.67 µg/g Cr for women. According to a 1967 survey, the average urinary Cd concentration of Jinzu River basin residents was as high as 19.8 µg/g Cr in men and 26.4 µg/g Cr in women, and that of the patients with *Itai-itai* disease was >30 µg/g Cr [28]. In the 1983–1984 and 1994–1995 health surveys of residents of the Jinzu River basin, the average urinary Cd concentrations were 13.3–13.5 µg/g Cr, and 7.5–7.7 µg/g Cr, respectively [29]. By contrast, the urinary Cd concentration in Cd non-polluted areas in Japan is 1–2 µg/g Cr [30–34]. The Cd concentrations of the residents of the Jinzu River basin after soil restoration were similar to those of residents of non-polluted areas.

In this study, the urinary Cd concentration was higher with age. Similarly, the level of urinary β2MG and NAG, markers of Cd-induced renal tubular damage, were higher in older subjects, and in those with a history of residence in the Cd-polluted area before soil restoration. A study conducted in the Kakehashi River basin in Ishikawa Prefecture reported that urinary Cd excretion was higher with Cd exposure of >30 years in men and >20 years in women [35]. After the soil restoration conducted in Kakehashi River basin in 1981, some residents were followed up to assess their health status, and it was found that urinary Cd concentrations did not decrease even 20–30 years after soil restoration [36–40]. The high urinary Cd concentrations in the elderly after soil restoration are likely due to high accumulation of Cd in the body due to long-term Cd exposure. Moreover, Cd accumulated in organs is excreted in urine due to organ destruction associated with aging and disease. Similarly, a follow-up study of residents of Kakehashi River basin reported that renal tubular dysfunction progressed even after soil restoration [36–40]. In this study, years of residence before soil restoration had significant relationships with urinary Cd concentration and markers for renal tubular function, independent of age. The levels of Cd and markers of renal tubular dysfunction were not high among the residents of the area after soil restoration, suggesting that soil restoration was effective. However, long-term Cd exposure due to residence prior to soil restoration may have caused renal tubular dysfunction even after soil contamination was reduced.

To clarify how soil restoration affects human health, we investigated the relationships among residence history before and after soil restoration, urinary Cd concentration, and markers of renal tubular function among people who were residents of Cd-polluted areas before soil restoration. In both men and women, urinary Cd concentration increased by 3% when years of residence before soil restoration increased by 5 years, and decreased by 3% when years of residence after soil restoration increased by 5 years. The effect of residence history in the Cd-polluted area on urinary β2MG was greater than that on urinary Cd: the urinary β2MG concentration increased by 7–10% when years of residence before soil restoration increased by 5 years and decreased by 6–9% when years of residence after soil restoration increased by 5 years. These results indicate that years of residence before soil restoration was related to the deterioration of health associated with Cd exposure, and that years of residence after soil improvement was related to improvement of health, *i.e.*, soil restoration improved the health of the residents of the formerly Cd-polluted area. However, renal dysfunction due to Cd exposure progresses over time, and lesions progress in patients with renal tubular dysfunction even when Cd exposure is reduced [41, 42]. Careful follow-up of renal function is needed in patients with renal tubular dysfunction who resided in Cd-polluted areas long before soil restoration.

The relationship between urinary NAG concentration and years of residence was weaker than that for β2MG in this study, and no significant relationship was observed between NAG concentration and years of residence before and after soil restoration among people who were residents of the Cd-polluted area before soil restoration. Urinary NAG and β2MG are used as markers of renal tubular dysfunction due to Cd exposure. β2MG is a low-molecular-weight protein (molecular weight = 11,800 Da) that freely passes through the glomerulus; 99.8% is reabsorbed by proximal renal tubules [43]. In patients with renal tubular dysfunction, impaired reabsorption increases urinary excretion. By contrast, NAG catalyzes glycoproteins, glycolipids, and glycosaminoglycans [44]. This enzyme is found in intracellular lysosomes in a variety of tissues and fluids. Its molecular weight is 100–140 kDa and it does not pass through the glomerulus. In the kidney, it is expressed mainly in proximal tubular epithelial cells and is excreted in very small amounts in urine by exocytosis. In patients with renal proximal tubular dysfunction, urinary excretion increases due to the destruction of renal tubular epithelial cells. Urinary NAG excretion increases from the early stage of renal damage even when urine protein is negative, and decreases as renal damage progresses because the renal tubular epithelium decreases. In this study, the correlation coefficient between NAG and β2MG was 0.41 for men and 0.49 for women, reflecting weak correlations. It is possible that NAG decreased even though urinary β2MG increased in patients with severe renal dysfunction who had long resided in the Cd-polluted area before soil restoration. This may explain the weak correlation between NAG and β2MG, and the lack of a significant relationship between years of residence and NAG.

The main strength of this study was the large scale of the survey of residents of a former Cd-polluted area. Previous studies focused on changes in health status in participants with a history of residence in Cd-polluted areas [36–40]. In this study, by examining the residents of a formerly Cd-polluted area, we evaluated the health status of people with no history of living in a Cd-contaminated area before soil restoration. In addition, we evaluated years of residence in the Cd-polluted area before and after soil restoration. A limitation of this study was the low participation rate; it is assumed that about 20% of the local general population participated. Another limitation was that the status of Cd exposure was evaluated only by years of residence. Even in Cd-polluted areas, the degree of Cd pollution differs depending on the location. Similarly, it is possible that people were exposed to Cd in food even if they lived outside the Cd-polluted area. Third, the cause of renal tubular dysfunction was not evaluated sufficiently. In this study, smoking was considered a confounding factor, but it was not possible to consider the history of diseases such as diabetes, hypertension, and chronic kidney disease because we lacked the necessary data. However, because the participants were residents of Cd-polluted areas, and because there were correlations between urinary Cd and markers of renal tubular dysfunction, the renal tubular defunction of the participants may have been caused by Cd exposure.

## Conclusions

We evaluated the health status of the general population of a former Cd-polluted area of the Jinzu River Basin. The urinary Cd concentrations of people with no history of residence prior to soil restoration were similar to those of people in Cd non-polluted areas. In addition, urinary Cd and β2MG, which are indices of renal tubular damage, were positively related to years of residence before soil restoration, and negatively related to years of residence after soil restoration, independent of age. These results suggest that Cd exposure and associated renal tubular dysfunction were influenced by Cd exposure from the environment prior to improvement of soil Cd contamination. Soil restoration is an effective measure to reduce the exposure to Cd of those loving in Cd-polluted areas.

## Abbreviations

Cd: cadmium
β2MG: β2-microgloblin
NAG: N-acetyl-β-D-glucosaminidase
Cr: creatinine
ICP-MS: inductively coupled plasma mass spectrometry
SE: standard error
CI: confidence interval

## Declarations

### Availability of data and material

Data available on request from authors.
